# Determinants of physician attitudes towards the new selective measles vaccine mandate in Germany

**DOI:** 10.1186/s12889-021-10563-9

**Published:** 2021-03-22

**Authors:** Julia Neufeind, Cornelia Betsch, Vera Zylka-Menhorn, Ole Wichmann

**Affiliations:** 1 Immunization Unit, Robert Koch Institute, Berlin, Germany; 2 Charité University Medicine Berlin, Berlin, Germany; 3 Center for Empirical Research in Economics and Behavioral Sciences (CEREB), University of Erfurt, Erfurt, Germany; 4 Media and Communication Science, University of Erfurt, Erfurt, Germany; 5 Deutsches Ärzteblatt, Cologne, Germany

**Keywords:** Vaccine hesitancy, Measles, Mandatory vaccination, Germany, Health care workers

## Abstract

**Background:**

In Germany, a mandatory policy on measles vaccination came into effect in March 2020. Physicians, as the main vaccine providers, have a crucial role in implementing it. Mandatory vaccination changes the preconditions under which patient-provider communication on vaccines occurs. Physicians might or might not favor vaccine mandates depending on, among other factors, their attitudes towards vaccines and capabilities as vaccine providers. The aim of this study was to investigate in different subgroups of physicians the association between various factors and their attitudes towards a mandatory policy.

**Methods:**

In total, 2229 physicians participated in a mixed-mode online/paper-pencil survey. Respondents were general practitioners, pediatricians, gynecologists, and internists. Primary determinants were the 5C psychological antecedents of vaccination, communication self-efficacy, patient clientele, projected consequences of the mandate and sociodemographic characteristics. Associations between outcomes and determinants were examined using linear regression analysis.

**Results:**

Approximately 86% of physicians were in favor of the measles vaccine mandate for children. Regarding the 5C model, physicians were more in favor of vaccine mandates when they scored higher on confidence and collective responsibility, and lower on complacency and calculation. They were more in favor of vaccine mandates when they had higher communication self-efficacy and a more vaccine-positive patient clientele. Pediatricians were less in favor of mandates for children (80.0%) than other physician subgroups (87.1%). They were also less convinced that a mandate would result in more children getting vaccinated (59.3%) than other physician subgroups (78.3%). When controlled for these expected consequences, being a pediatrician no longer lowered the attitude towards the mandate.

**Conclusions:**

Physicians in Germany are predominantly in favor of a measles vaccine mandate. Whether or not physicians believe the mandate to be effective in increasing vaccine coverage affects their attitude towards the mandate. In pediatricians, this belief explains their less positive attitude towards the mandate. In addition, physicians need adequate support to communicate well with patients, especially those who are hesitant, to booster their communication self-efficacy. To increase acceptance of vaccine mandates, the 5C model can be used, e.g., collective responsibility can be communicated, to avoid anger stemming from a negative attitude to mandates.

**Supplementary Information:**

The online version contains supplementary material available at 10.1186/s12889-021-10563-9.

## Background

In March 2020, as part of the Measles Protection Act, a new mandatory policy on measles vaccination came into effect in Germany, requiring proof of measles immunization for all children and staff in childcare and schools, as well as health workers [1]. If individuals are not able to provide this proof, a variety of sanctions can be implemented: access to pre-school childcare can be rejected, at school penalties up to EUR 2500 can be collected, new employment can be rejected, and employees can be deployed elsewhere. Exemptions exist for individuals with proof of naturally-acquired immunity or with medical contraindications, e.g., allergy to vaccine components. The level of enforcement of the mandate has yet to be evaluated. This is the first vaccine mandate to be implemented in Germany, at least when only considering the last decades. Historically, there have been vaccine mandates, in the 19th and beginning of the twentieth century, against smallpox and in the German Democratic Republic against a variety of diseases.

In the context of the 2018 European Council recommendation to take action against vaccine hesitancy [2], the World Health Organization (WHO) listing vaccine hesitancy as one of the ten threats to global health in 2019 [3], large outbreaks of measles in Germany and the European region [4], and new mandates in Italy [5] and France [6], discussion evolved regarding a mandatory measles vaccination policy in Germany. In the winter of 2019, the Measles Protection Act passed in the German parliament with several measures to improve the immunization system in Germany, including new regulations on disease notifications, submission of physician claims data to be included in an immunization information system, and the obligation to provide a proof of measles protection of children and specific professional groups.

Physicians in private practices have a key role in implementing this law in Germany. First and foremost, they are the ones who primarily provide vaccines to the child and adult populations in Germany and counsel patients on vaccines. Among private physicians, the main vaccine providers are pediatricians, general practitioners (GPs), internists, and gynecologists. Pediatricians provide vaccines mainly to children, while GPs, internists and gynecologists provide vaccines mainly to adults. Secondly, they are obliged by the new law to ensure that their staff is protected against measles as well. And lastly, they themselves are obliged to be vaccinated, if not born before 1970 or protected by naturally-acquired immunity. Hence, with regard to implementation, the legislator depends on provider cooperation in these three important ways. The Measles Protection Act changes the context in which physicians operate as vaccine providers. Understanding physician attitudes towards mandates is crucial if we want to understand how physicians will implement the mandate and how the mandate may affect other vaccine decisions [7]. In particular, concerns were raised that the mandate may evoke reactance among physicians who might in turn be less likely to recommend other voluntary vaccines to their patients [8].

Physicians differ in attitude towards vaccinations and these differences affect their recommendations and vaccination behaviors [9, 10]. In addition, physicians might differ in their attitudes towards mandates. A mandate drastically reduces free choice regarding vaccination decisions for their patients, their staff, and themselves. Physicians, who formerly had the task to convince patients of measles vaccination, can now refer to the mandate. This might make it easier for those who did not feel confident before in their ability to talk to patients about the vaccines and explain their value. It might also relieve physicians who face many vaccine-hesitant patients, as they can now refer to the mandate. Mandates can also set new social norms or foster pre-existing norms, thus interacting with physician attitudes towards vaccines [8]. Physicians base their own vaccine decisions, and to some extent, their vaccine recommendations, on their own confidence in vaccines and the system that delivers them, their collective responsibility (willingness to protect others), constraints (perceived barriers), complacency (not perceiving diseases as a high risk), and calculation (engagement in extensive information search). These 5C psychological determinants of vaccination behavior [11] might also be associated with physician endorsement of vaccine mandates.

We conducted a national survey among private physicians in Germany shortly before the mandate became effective. Based on the above-mentioned considerations, we first explored whether physician attitude to the mandate was associated with the 5C psychological determinants of vaccination behavior. Secondly, we explored whether communication self-efficacy and patient clientele, as well as the expected consequences of the mandate, would be associated with attitude towards the mandate.

## Methods

### Study population recruitment

We pursued a mixed-mode design as an online and paper questionnaire. The survey was sent out as a paper-pencil survey to 90,000 private physicians as a supplement to Deutsches Ärzteblatt, the major medical journal in Germany that is provided free of cost to all physicians. The distribution of the questionnaire was limited to GPs, internists, pediatricians, and gynecologists – the primary vaccine providers in Germany - but also occupational physicians, dermatologists, and neurologists. On the survey, a QR code and link to the online survey was provided for convenience. In addition, a link to the online survey was sent via e-mail newsletter to subscribers of Deutsches Ärzteblatt. It is noteworthy that participation via newsletter did not require identification as a physician. In addition, the link to the online survey was posted on the respective website of the journal (for registered users). Data was obtained from January 24 to March 6, 2020. As an incentive, participants could opt into a lottery with the chance to win a tablet computer or a stethoscope.

### Survey instrument

All items used (translated into English), the R code and complete survey, can be found here (https://osf.io/pbgef/). Our primary study outcome was the attitude towards vaccine mandates, assessed by four self-developed items. These included items on attitudes towards a selective measles mandate for children and health care personnel, and towards a general mandate for all vaccines recommended for children, e.g., ‘the measles vaccine should be mandatory for children in school and kindergarten’. For each of these items, respondents stated their level of agreement on a five-point-Likert-scale ranging from ‘strongly disagree’ (score = 1) to ‘strongly agree’ (score = 5). We calculated a mean score for ‘attitude towards mandates’, consisting of four items (Cronbach’s alpha = 0.88), ranging from a negative attitude towards the mandate (score = 1) to a positive attitude (score = 5). In addition, we developed items to assess physicians’ projected consequences of the Measles Protection Act in their practice, e.g., ‘the mandate will be a burden for the patient-provider relationship’. For each of these items, the respondents stated their level of agreement on a five-point-Likert-scale ranging from ‘strongly disagree’ (score = 1) to ‘strongly agree’ (score = 5). The 5C short scale on psychological determinants contained five items, one for each construct (confidence, collective responsibility, constraints, complacency and calculation), e.g., ‘I am completely confident that vaccines are safe’. For each of these items the respondents stated their level of agreement on a five-point-Likert-Scale ranging from ‘strongly disagree’ (score = 1) to ‘strongly agree’ (score = 5) [11].

We assessed physician confidence in communicating with patients about vaccines using self-efficacy items, e.g., ‘how confident are you in your ability to talk with patients and parents about vaccines?’ [12]. Respondents stated their level of confidence on a five-point-Likert-scale ranging from ‘not at all confident’ (score = 1) to ‘very confident’ (score = 5). We calculated a mean score for ‘communication self-efficacy’, consisting of four items (Cronbach’s alpha = 0.87).

Four items from a knowledge scale assessed the level of misinformation, e.g., ‘vaccinations increase the occurrence of allergies’ (possible answers: agree, disagree, or don’t know) [13]*.* We calculated a sum score for ‘vaccine knowledge’, in which every correct answer was counted as one point and every false answer or don’t know answer was counted as zero points.

We quantified patient positions on vaccination, i.e., the patient clientele, drawing on a taxonomy introduced by Leask et al. [14]. Accordingly, patients can broadly be divided into (i) unquestioning acceptors, (ii) cautious acceptors, (iii) late/selective vaccinators, and (iv) refusers. After briefly describing the characteristics, e.g., ‘what is the proportion of your patients or parents who accept vaccines without questions?’, we asked participants to estimate what portion of patients would fall into each category (reported as %).

Sociodemographic characteristics collected included occupational group (i.e., GP, pediatrician, gynecologist, or internist), gender, years of work experience, region (i.e., eastern or western Germany), and city size.

Beyond the measles vaccine mandate, a variety of measures to increase vaccine uptake have been discussed in Germany. As part of the Measles Protection Act, the option to pilot test the provision of influenza vaccination in pharmacies has been introduced in some regions in Germany, starting with the 2020–21 season. We asked participants whether they were in favor of the following measures (possible answers: yes, no, or don’t know): vaccination in schools, vaccination in pharmacies, and introduction of a digital vaccination card for their patients.

### Statistical analyses

Analyses were conducted in R [15]. Agreement to items was measured using a five-point-Likert-scale, and descriptive data was reported as: percentage who disagreed (= 1, 2), were undecided (= 3) or agreed (= 4, 5). Complete case analysis was pursued for all items. We performed blockwise multiple linear regressions to identify correlates of the attitude towards vaccine mandates. Model one contained sociodemographic characteristics, i.e., work experience, gender, region, city size, and occupational group. In the second step, ‘communication self-efficacy’ and ‘patient clientele’ were added. In the third step, the 5C psychological determinants of vaccination were added. In the fourth step, the expected consequence (‘more children vaccinated’) were added. We report β estimates, 95% confidence intervals (CI), and R^2^ to assess model fit. We computed variance inflation factors (VIF) to test for multicollinearity and interpreted values < 5 as presenting no multicollinearity issues (there was no issue of multicollinearity in our regression models). Next, we tested for mediation of these relationships using the mediation package [16]. Our model included occupational group as the predictor variable, expected consequences of the mandate as the mediator variable, and attitude towards the mandate as the outcome variable. We further assessed differences between pediatricians and other physician subgroups using t-tests. We assessed correlations between the attitude towards the mandate and projected consequences using Pearson’s method.

## Results

### Response and sociodemographic characteristics

In total, 2762 physicians participated in the survey. Of these, 2467 indicated belonging to the initial survey target groups (i.e., GPs, gynecologists, internists, and pediatricians). Other medical specialists, excluded from analysis, totalled 295 of the participants. We further excluded 238 participants who received survey invitations via newsletter. Their answers differed significantly from physicians receiving other modes of invitation. Specifically, participants who used the newsletter link were significantly more vaccine hesitant (confidence, complacency) and had a high proportion of missing data (approximately 20% per variable). This led to the assumption this mode had been taken over by vaccine deniers. Furthermore, and in contrast to all other ways to enter the survey, there was no way to ensure that the participating individuals were indeed physicians. To ensure high data quality, we eventually included 2229 participants in our analysis (1140 participated via online survey and 1089 via paper-pencil survey). Among these, 1178 were GPs, 259 gynecologists, 416 internists, and 376 pediatricians. Missing values were below 5% for all items. With our final dataset, we assessed potential mode effects in our regression model, including mode of participation (paper vs. online) as a covariate, and found no significant mode effect. On average, respondents had 19 years of work experience and 54.3% were female. For further characteristics of the study population, see Table 1.

**Table 1:** Characteristics of study population

Variable	Level	Pediatrician	GP	Gynecologist	Internist	***p***-value
n		376	1178	259	416	
Gender: n (%)	Male	173 (48.2)	512 (46.0)	63 (25.6)	221 (55.1)	< 0.001
	Female	186 (51.8)	601 (54.0)	183 (74.4)	180 (44.9)	
Work experience: mean (SD)		18.68 (10.38)	20.25 (10.97)	19.35 (10.47)	16.50 (9.79)	< 0.001
Region: n (%)	Western	283 (80.2)	895 (81.4)	184 (75.7)	321 (80.2)	0.260
	Eastern	70 (19.8)	205 (18.6)	59 (24.3)	79 (19.8)	
City size: n (%)	< 10.000	41 (11.5)	397 (35.7)	27 (11.1)	64 (15.9)	< 0.001
	10.000–100.000	181 (50.8)	428 (38.5)	123 (50.4)	192 (47.6)	
	> 100.000	134 (37.6)	286 (25.7)	94 (38.5)	147 (36.5)	
Attitude towards mandates: mean (SD)^a^		4.00 (0.93)	4.03 (0.97)	4.32 (0.82)	4.19 (0.92)	< 0.001
Reported patient clientele: mean (SD)	Unquestioning acceptor	72.73 (20.49)	61.23 (22.94)	49.63 (24.25)	60.45 (20.68)	< 0.001
	Cautious acceptor	19.03 (16.47)	22.60 (16.28)	28.10 (16.62)	21.91 (14.89)	< 0.001
	Selective vaccinator	5.51 (6.58)	11.44 (11.04)	14.36 (13.14)	11.91 (10.18)	< 0.001
	Refuser	2.73 (6.26)	4.73 (5.92)	7.92 (8.66)	5.73 (5.78)	< 0.001
Communication self-efficacy: mean (SD)^b^		4.49 (0.58)	4.24 (0.63)	4.26 (0.61)	4.22 (0.66)	< 0.001
Vaccine knowledge: mean (SD)^c^		3.85 (0.50)	3.61 (0.86)	3.67 (0.73)	3.70 (0.70)	< 0.001

^a^Mean score ‘attitude towards mandates’ consisting of four items (Cronbach’s alpha = 0.88) expressing negative attitude (score = 1) to positive attitude (score = 5)

^b^Mean score ‘communication self-efficacy’ consisting of four items (Cronbach’s alpha = 0.87) ranging from very low (score = 1) to very high (score = 5)

^c^Sum score ‘vaccine knowledge’ consisting of four items, every correct answer was counted as 1 point and every false answer or ‘don’t know’ answer was counted as 0 points

### Attitude towards vaccine mandates, expected consequences of the measles mandate, and vaccine knowledge

Of the participants, 85.9 and 88.2% of physicians agreed that the measles vaccine should be mandatory for children and health care workers, respectively (5.4% and 5.1 undecided, 8.7 and 6.8% disagreed, respectively). It is noteworthy that pediatricians were less in favor of a mandate for children (80.0%, mean [M] = 4.2) than other physician subgroups (87.1%, M = 4.4, t [489] = − 3.3, *p* <  0.001); however, they were more in favor of a mandate for health care workers (92.2%, M = 4.6) than other physician subgroups (87.4%, M = 4.4, t [614] = 5.0, *p* <  0.001). Seventy percent of participants indicated that all recommended vaccines for children should be mandatory (13.3% undecided, 16.7% disagreed). Of the participants, 16.8% agreed that everybody should be able to decide freely about themselves and their children (17.4% undecided, 65.8% disagreed). The latter item was reverse-coded to build the mean score. Pediatricians were less in favor that all recommended vaccines for children should be mandatory (61.0%, M = 3.6) than other physician sub-groups (71.8%, M = 3.9, t [497] = − 4.1, *p* < 0.001) and more in favor that everybody should be able to freely decide about themselves and their children (21.1%, M = 2.4) than other physician subgroups (15.8%, M = 2.3, t [497] = 2.0, *p* < 0.05).

The attitude towards mandates was predominantly associated with the projected consequences of the mandate for their own practice (Table 2). The strongest correlation was found for the following two items: (i) the more physicians expected that due to the new law more children would be vaccinated on time, the more they had a positive attitude towards mandates, and (ii) the more physicians expected the mandate to be a burden for the patient-provider relationship, the more they had a negative attitude towards mandates.

**Table 2:** Expected consequences of the mandate and correlation with attitude towards mandates for pediatricians vs. others

Expected consequences of mandate	Mean (SD)^a^			Correlation^b^ with attitude towards mandates^c^	
	Pediatricians	Other physicians	*p*-value	Pediatricians	Other physicians
I expect no consequences.	**2.97 (1.29)**	**3.16 (1.33)**	0.014	**0.11**	**0.09**
Counseling patients will require more effort.	**3.01 (1.13)**	**3.23 (1.09)**	0.001	**−0.16**	**− 0.15**
Counseling patients will require less effort.	2.01 (0.96)	1.94 (0.87)	0.203	**0.17**	**0.12**
The mandate will be a burden for the patient provider relationship.	**2.11 (0.95)**	**1.98 (0.97)**	0.018	**−0.34**	**− 0.35**
I expect a higher amount of work for issuing certificates about measles protection to patients.	**2.47 (1.12)**	**2.33 (1.00)**	0.017	**−0.19**	**−0.24**
I expect that patients will press me to issue medical exemptions from the mandate.	**3.38 (1.24)**	**3.16 (1.26)**	0.003	−0.04	**−0.19**
I expect more children to be vaccinated on time.	**3.47 (1.06)**	**3.93 (0.90)**	< 0.001	**0.25**	**0.35**

Bold denotes significance at *p* < 0.05.

^a^Likert scale items (1 = disagree; 5 = strongly agree)

^b^Pearson’s method

^c^Mean score ‘attitude towards mandates’ consisting of four items (Cronbach’s alpha = 0.88) expressing negative attitude (score = 1) to positive attitude (score = 5)

Pediatricians predominantly expected more negative consequences of the mandates for their own practice than other physician subgroups (Table 2). The largest difference was found for the expected vaccine uptake, as significantly less pediatricians (59.3%) than other physician subgroups (78.3%) expected more children to be vaccinated on time. Also, significantly more pediatricians than other physician subgroups expected the mandate to be a burden for the patient-provider relationship. Among participants, there was a wide spread of opinions on whether or not they expected consequences from the mandate for their own practice (44.1% agreed, 20.3% were undecided, 35.7% disagreed). Similarly, physicians were divided on whether or not counseling patients would require more effort (46.3% agreed, 23.3% undecided, 30.4% disagreed). Only 7.1% indicated that the mandate would lead to less effort in vaccine counseling (13.3% undecided, 79.5% disagreed). Of the participants, 48.2% expected a higher amount of work for issuing certificates on measles protection to patients (17.1% undecided, 34.7% disagreed), and 16.0% expected that patients would press them to issue medical exemptions from the mandate (18.8% undecided, 65.2% disagreed).

With regard to vaccine knowledge, 79.6% of participants answered all items correctly. Only among 1.3% of participants were all answers false. On average, participants had scores of 3–4 out of 4 correct answers (M = 3.6, standard deviation [SD] =0.77). Vaccine knowledge differed significantly among occupational groups (Table 1), with pediatricians exhibiting more knowledge (M = 3.85) than other physician sub-groups (M = 3.64, t [803] = 6.7, *p* < 0.001).

### Correlates of the attitude towards the mandatory policy

Table 3 presents results of a series of stepwise regressions predicting the attitude towards the measles mandate. Region, work experience, occupational group, communication self-efficacy, patient clientele, the 5C psychological determinants, and whether more children were expected to be vaccinated on time were associated with attitude towards mandates. Physicians from eastern Germany and those with more years of work experience had more positive attitudes towards vaccine mandates. The higher the communication self-efficacy and the more patients were unquestioning acceptors, the more physicians had positive attitudes towards vaccine mandates. Physicians had more positive attitudes towards the vaccine mandate the higher their confidence or collective responsibility, and the lower their complacency or calculation. Being a pediatrician was associated with a more negative attitude towards mandates. The coefficients increased when communication self-efficacy and patient clientele were added to the model and remained stable when the 5C determinants were added. When the expected consequence ‘more children vaccinated’ was added, the coefficients became insignificant (GPs and internists) or decreased (gynecologists), i.e., the degree to which participants expected the mandate to increase vaccine uptake among children, and provided an explanation for the difference in attitude towards the mandate between physician subgroups. We explored this effect in mediation analysis.

**Table 3:** Multiple linear regression models for attitudes towards vaccine mandates^a^

		Model 1	Model 2	Model 3	Model 4
Explanatory variables		β (95% CI)	β (95% CI)	β (95% CI)	β (95% CI)
(Intercept)		3.80 (3.64–3.95)	2.45 (2.11–2.80)	1.52 (0.92–2.11)	0.91 (0.33–1.49)
**Region**	Western	*Reference*	*Reference*	*Reference*	*Reference*
	Eastern	**0.26 (0.16–0.37)**	**0.25 (0.15–0.35)**	**0.23 (0.14–0.33)**	**0.26 (0.16–0.35)**
**Gender**	Male	*Reference*	*Reference*	*Reference*	*Reference*
	Female	0.05 (− 0.03–0.14)	**0.09 (0.01–0.18)**	0.04 (− 0.04–0.12)	− 0.00 (− 0.08–0.07)
**City size**	< 10.000	*Reference*	*Reference*	*Reference*	*Reference*
	10.000–100.000	−0.03 (− 0.14–0.07)	−0.01 (− 0.12–0.09)	−0.02 (− 0.11–0.08)	−0.01 (− 0.11–0.08)
	> 100.000	− 0.03 (− 0.15–0.08)	−0.02 (− 0.13–0.09)	−0.02 (− 0.12–0.08)	−0.04 (− 0.14–0.05)
**Work experience**		**0.01 (0.00**–**0.01)**	**0.01 (0.00**–**0.01)**	**0.01 (0.00**–**0.01)**	**0.01 (0.00**–**0.01)**
**Occupational group**	Pediatrician	*Reference*	*Reference*	*Reference*	*Reference*
	GP	0.01 (−0.10–0.13)	**0.13 (0.01**–**0.24)**	**0.14 (0.04**–**0.25)**	0.02 (−0.08–0.13)
	Gynecologist	**0.30 (0.14**–**0.45)**	**0.45 (0.29**–**0.61)**	**0.33 (0.18**–**0.47)**	**0.19 (0.04**–**0.33)**
	Internist	**0.20 (0.06**–**0.33)**	**0.31 (0.18**–**0.45)**	**0.25 (0.12**–**0.37)**	0.11 (−0.01–0.24)
**Communication self-efficacy**^b^			**0.22 (0.16**–**0.29)**	**0.11 (0.05**–**0.18)**	**0.10 (0.04**–**0.16)**
**Patient clientele**^c^			**0.45 (0.27**–**0.64)**	0.16 (− 0.01–0.33)	0.14 (− 0.02–0.31)
**Confidence**				**0.40 (0.34**–**0.46)**	**0.35 (0.29**–**0.41)**
**Collective responsibility**				**0.10 (0.02**–**0.19)**	**0.12 (0.04**–**0.20)**
**Constraints**				−0.04 (−0.09–0.01)	−0.04 (− 0.09–0.01)
**Complacency**				**−0.32 (− 0.42**– **− 0.22)**	**−0.28 (− 0.38**– **− 0.18)**
**Calculation**				**−0.09 (− 0.12– − 0.06)**	**−0.09 (− 0.11**– **− 0.06)**
**More children vaccinated**^d^					**0.25 (0.21**–**0.29)**
Observations		1974	1974	1974	1974
R^2^/R^2^ adjusted		0.034/0.030	0.069/0.064	0.219/0.213	0.277/0.271

Blockwise inclusion of covariates in Model 1, Model 2, and Model 3

Bold denotes significance at *p* < 0.05

^a^Mean score ‘attitude towards mandates’ consisting of four items (Cronbach’s alpha = 0.88) expressing a negative attitude (score = 1) to a positive attitude (score = 5)

^b^Mean score ‘communication self-efficacy’ consisting of four items (Cronbach’s alpha = 0.87) ranging from very low (score = 1) to very high (score = 5)

^c^‘Patient clientele’ as portion of patients who are unquestioning acceptors of vaccination ranging from 0 (0%) to 1 (100%)

^d^Expected consequence of the mandate, item: ‘I expect more children to be vaccinated on time’ (1 = disagree; 5 = strongly agree)

**Fig. 1:**
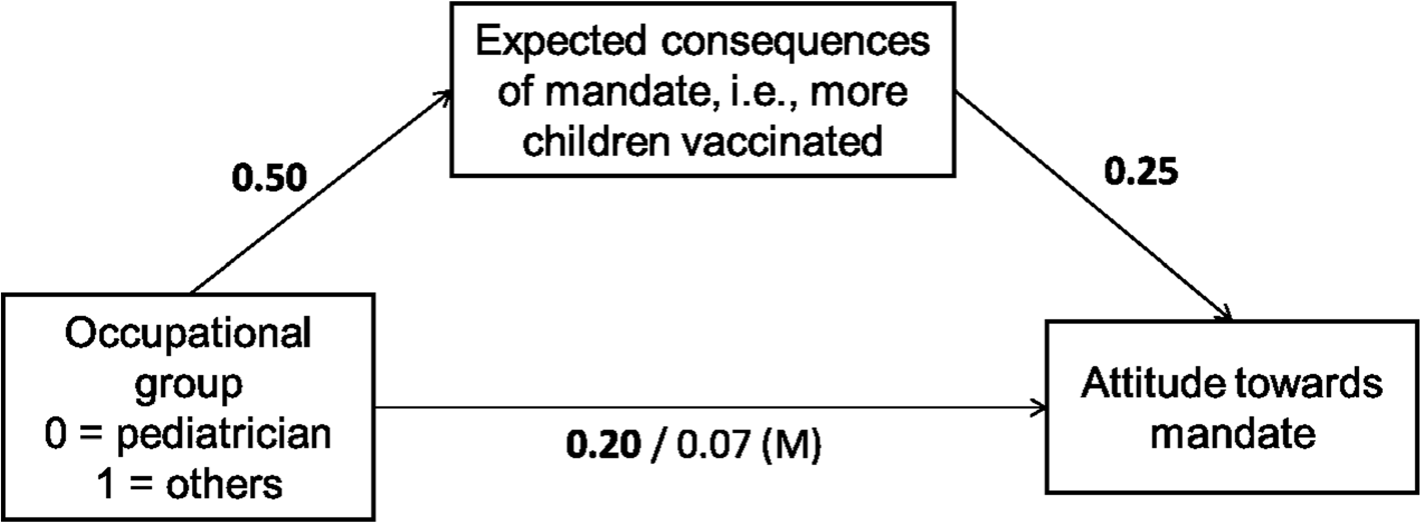
Mediation analyses. *Note:* All coefficients are β coefficients. Bold denotes significant at *p* < 0.05. The path coefficients after the slash indicate the relation between the occupational group and attitude towards mandate controlled for expected consequences. (M) indicates a significant mediation effect. Covariates include sociodemographic characteristics, communication self-efficacy, patient clientele and 5C psychological determinants

Figure 1 depicts the mediation model used to test whether the expected consequences of the mandate for their own practice mediate the effect of occupational group on the attitude towards the mandate (see Supplementary Table 1 also). We included sociodemographic characteristics, communication self-efficacy, patient clientele, and the 5C determinants as covariates. Not being a pediatrician significantly increased the attitude towards the mandate, which was completely mediated by an increased belief in expected consequences for their own practice, i.e., more children being vaccinated on time (Average Causal Mediation Effect [ACME]: β = 0.13, 95% CI = 0.09–0.17, *p* < 0.05). Thus, a complete mediation effect occurred. When controlling for the expected consequences, being a pediatrician no longer lowered the attitude towards the mandate.

Two-thirds of physicians were in favor of vaccination programs in schools (61.5% endorsed, 30.9% rejected, 7.6% undecided). Only 4.3% endorsed the vaccination in pharmacies (91.8% rejected, 3.9% undecided). The majority endorsed the introduction of a digital vaccination cards (58.1% endorsed, 21.7% rejected, 20.2% undecided).

## Discussion

The Measles Protection Act was initiated by the Ministry of Health in May 2019 in a political and societal climate of broad acknowledgement that something had to be done to strengthen the national immunization program and to fight vaccine hesitancy in Germany [17]. Former plans to eliminate measles by 2015 had failed [18]. Vaccination coverage for children was high (> 95% MCV1), but vaccination was often delayed and incomplete [19]. Furthermore, considerable vaccination gaps were identified among young adults (80% coverage for adults aged 18–29 years, 47% for those aged 30–39 years) [20]. A mandate was considered to be one option among others to increase vaccine uptake and as such both the governing parties and medical societies as well as other stakeholders supported it [21–23]. Some stakeholders, however, expressed concerns regarding the legal, sociological and ethical dimension of such a mandate, e.g. the German Ethics Council [24]. Moreover, potential psychological consequences were discussed, such as detrimental effects on the willingness to vaccinate against diseases where vaccination remained voluntary [25–27]. The result of the controversy was a law that encompassed - beyond a mandate - different aspects to strengthen the immunization system in Germany [1]. This study aimed at understanding physicians’ attitudes towards vaccine mandates shortly before the introduction of the law.

A large majority of the private physicians who participated in our survey were in favor of the new measles vaccination mandate and had a positive attitude towards vaccine mandates in general. However, pediatricians, i.e., those physicians who primarily vaccinate children against measles, were less in favor of a measles vaccine mandate compared to other physician subgroups. The more participants expected negative consequences of the mandate for their own practice, e.g., more work or a burden for patient-provider relationships, the more they had negative attitudes towards mandates.

We assumed that physicians who regularly encountered difficulties in vaccine counseling would have a positive attitude towards the mandate, as the mandate might eliminate their role in discussing the rationale for the measles vaccine. However, in our study, we observed the contrary. Physicians had a more negative attitude towards the mandate when they had lower confidence in communicating with patients about vaccines, i.e., lower communication self-efficacy and a lower proportion of unquestioning acceptors among their patients. In contrast, physicians who felt higher communication self-efficacy and who had more unquestioning acceptors among their patient clientele were more likely to endorse vaccine mandates.

Among the psychological determinants, a more negative attitude towards the mandate was associated with lower vaccine confidence, lower collective responsibility, higher complacency, and higher calculation, while no effect was found for constraints. With regard to confidence, we assume that when physicians question vaccine safety, mandatory vaccines appear worrisome, as vaccination may put the vaccinated person at risk for potentially adverse events. From an ethical perspective, a mandate violates individual liberty, i.e., free will, but this violation may be justified as long as the mandate maximizes individual health. Those who lack confidence in vaccine safety, however, may question this benefit to individual health [28]. Likewise, a French study conducted shortly before the implementation of new vaccine mandates in France found fear of side-effects to be associated with a more negative attitude towards mandates among the general population [29]. In an Italian study pregnant women were more likely to favor vaccine mandates when they felt that health professionals were honest to them about the risk of vaccines [30]. With regard to collective responsibility, we believe that physicians who vaccinate themselves and others in order to protect others could favor a mandate, because a mandate restricts individual liberty in order to protect others. In contrast, physicians who vaccinate for self-protection only (low collective responsibility) will be more willing to take advantage when enough others are vaccinated and thus, might oppose a mandate, as their personal risk of infection is relatively low [31]. In accordance with this, an Australian study among health care workers found that the protection of co-workers was among the primary reasons for supporting an influenza vaccine mandate [32]. With regard to complacency, we suggest that when risk perception is high enough, more drastic measures seem acceptable. The above-mentioned French study found that the perception that vaccines bring important health benefits was associated with a more positive attitude towards mandates [29]. In our study, participants with high calculation were more likely to have a negative attitude towards vaccine mandates. Individuals with high calculation base their decisions on utility maximization, i.e., engaging in extensive information seeking and attempting to make the best decision for themselves [8]. In previous studies, it was shown that calculation is associated with non-vaccination [11]. Physicians high on calculation might see their freedom to take these selfish, rational decisions infringed by a vaccination mandate [26].

Pediatricians had more negative attitudes towards vaccine mandates (especially mandates for children) than other physician subgroups. Pediatricians were also less confident that more children would be vaccinated due to the mandate than other physician subgroups. Thus, those physicians who have the most experience in vaccinating children were less in favor of a measles mandate for children and less convinced it would successfully increase coverage. We found that not being a pediatrician increased the perception that more children would be vaccinated (expected consequence), which in turn increased a positive attitude towards mandates (mediation effect). At the same time, pediatricians were better informed about vaccine safety (vaccine knowledge), and had a higher communication self-efficacy and more unquestioning acceptors among their patients.

Some limitations of our study need to be acknowledged. The survey does not control for social desirability. The survey was conducted, among others, on behalf of the national public health authority in Germany, which might have enhanced social desirability, possibly leading to an overestimation of a favorable attitude towards vaccine mandates and vaccines, in general. Since it is unknown how many physicians we reached with questionnaires (mixed-mode online/offline), we were not able to calculate a response rate. There might be a selection bias in that those more engaged in the topic, either pro-vaccine or vaccine hesitant, might have been more likely to participate. By incentivizing participation through a lottery, we tried to encourage participation and reduce selection bias. Due to our study design we were unable to determine whether our survey population is representative of Germany’s primary care providers. We could show, however, that the distribution of gender and region in our study population was similar to the distribution of gender and region among physicians in Germany (Supplementary Table 2) [33]. Furthermore, the means for the 5C psychological determinants of vaccination in our study (GPs only) were very similar to a comparable study among German family physicians which used random sampling (data collection 2017/18) [34].

There are few studies that evaluate determinants of attitudes towards vaccine mandates [35, 36]. A systematic review on attitudes towards vaccine mandates found varying degrees of approval of vaccine mandates in different countries and different populations. However, the authors conclude that few studies go beyond a mere description of approval rates to the respective mandates and suggest that further studies should investigate the determinants of attitudes towards mandates [37]. Our study contributes to filling this knowledge gaps. A follow-up study is planned for 2022 to assess whether physician attitudes towards vaccine mandates have changed and whether expected consequences occurred. As part of a larger research project evaluating the measles vaccine mandate in Germany, a longitudinal survey study among parents has been initiated in August 2020.

## Conclusions

Shortly before the introduction of a measles vaccine mandate in Germany, the majority of physicians were in favor of the mandate and vaccine mandates, in general. The attitudes, however, differed. This study identified determinants of these attitudes and hence has implications for policy and further research:

Pediatricians, even though well-versed vaccine providers, were more hesitant towards the mandates, especially for the group of patients they serve – children. Their lack of confidence in the ability of the mandate to increase vaccine coverage among children explains their hesitancy towards mandates. Given their high expertise as vaccine providers, their concern deserves further investigation. Evaluation of the vaccine mandate and its effects on vaccine uptake and vaccine hesitancy is needed - not only for this reason - to enable an evidence-based discussion. This includes investigation into other tools used by pediatricians to increase vaccine uptake (e.g. reminder systems).

In addition, negative attitudes on mandates occur among physicians with lower communication self-efficacy and higher numbers of vaccine hesitant patients. Therefore, physicians need adequate support to communicate well with patients, especially hesitant patients. This includes offering training for physicians in doctor-patient conversation, e.g. applying promising techniques such as motivational interviewing [38], and making vaccination more prominent in medical training at university.

Our study suggests that physicians who have low confidence in vaccination, low collective responsibility and high complacency have a more negative attitude towards mandates, and might react with anger, similar to an effect observed in patients [26, 27]. It remains unclear which consequences anger could have on both physicians and patients. Public health institutions can try to prevent anger by communicating herd protection, thus countering detrimental effects of vaccine mandates, e.g., lower vaccine uptake for other voluntary vaccines [27]. This means that– in all communication activities - vaccination is framed as a collective responsibility, elimination of measles as a common effort, and vaccination as a way to protect those who cannot protect themselves [39]. Furthermore, public health institutions should invest more in transparency concerning vaccine safety and effective communication of the risks of vaccine-preventable diseases, e.g., the resurgence of measles, if they want to maintain physician support in vaccine mandates. This involves making vaccine safety monitoring data more accessible to laypersons, and debunking vaccine safety myths [40].

Whether or not mandates are effective in increasing measles vaccine uptake in Germany is yet to be evaluated. Omer et al. have argued that mandates can be effective if implemented with care and consideration of context [25]. Physician support of mandates, however, cannot be taken for granted.

## Supplementary Information

**Additional file 1: Supplementary Table 1.** Title: Mediation analyses: Effect of occupational group (X) on attitude towards mandates (Y) via expected consequences (M). **Supplementary Table 2.** Title: Sociodemographic characteristics of the study population and of physicians in Germany

## Data Availability

The datasets used and analyzed during the current study are available from the corresponding author on reasonable request.

## Abbreviations

GP: General practitioner
ACME: Average Causal Mediation Effect
M: Mean
WHO: World Health Organization
SD: Standard deviation
CI: Confidence interval
VIF: Variance inflation factor

## References

[1] Bundesregierung (2019). [Draft of a law for the protection from measles and for the strengthening of prevention through vaccination (Masernschutzgesetz)] Entwurf eines Gesetzes für den Schutz vor Masern und zur Stärkung der Impfprävention (Masernschutzgesetz).

[2] The Council of the European Union (2018). Council recommendation of 7 December 2018 on strengthened cooperation against vaccine-preventable diseases. Off J Eur Union.

[3] WHO. Ten threats to global health in 2019 [https://www.who.int/news-room/spotlight/ten-threats-to-global-health-in-2019. Accessed 2 Nov 2020.

[4] Thornton J (2019). Measles cases in Europe tripled from 2017 to 2018. BMJ..

[5] D’Ancona F, D’Amario C, Maraglino F, Rezza G, Iannazzo S. The law on compulsory vaccination in Italy: an update 2 years after the introduction. Euro Surveill. 2019;24(26):1900371. 10.2807/1560-7917.ES.2019.24.26.1900371 PMC6607737 31266589

[6] Lévy-Bruhl D, Fonteneau L, Vaux S, Barret A-S, Antona D, Bonmarin I (2019). Assessment of the impact of the extension of vaccination mandates on vaccine coverage after 1 year, France, 2019. Eurosurveillance..

[7] MacDonald NE, Harmon S, Dube E, Steenbeek A, Crowcroft N, Opel DJ (2018). Mandatory infant & childhood immunization: rationales, issues and knowledge gaps. Vaccine..

[8] Betsch C, Fiske ST, Böhm R, Chapman GB (2015). Using behavioral insights to increase vaccination policy effectiveness. Policy Insights Behav Brain Sci.

[9] Betsch C, Wicker S (2014). Personal attitudes and misconceptions, not official recommendations guide occupational physicians' vaccination decisions. Vaccine..

[10] Betsch C, von Hirschhausen E, Zylka-Menhorn V. Professionelle Gesprächsführung – wenn Reden Gold wert ist. Deutsches Ärzteblatt. 2019;116(11):A-520 / B-427 / C-422.

[11] Betsch C, Schmid P, Heinemeier D, Korn L, Holtmann C, Böhm R (2018). Beyond confidence: development of a measure assessing the 5C psychological antecedents of vaccination. PLoS One.

[12] Henrikson NB, Opel DJ, Grothaus L, Nelson J, Scrol A, Dunn J, Faubion T, Roberts M, Marcuse EK, Grossman DC (2015). Physician communication training and parental vaccine hesitancy: a randomized trial. Pediatrics..

[13] Zingg A, Siegrist M (2012). Measuring people's knowledge about vaccination: developing a one-dimensional scale. Vaccine..

[14] Leask J, Kinnersley P, Jackson C, Cheater F, Bedford H, Rowles G (2012). Communicating with parents about vaccination: a framework for health professionals. BMC Pediatr.

[15] R Core Team (2016). R: A language and environment for statistical computing.

[16] Tingley D, Yamamoto T, Hirose K, Keele L, Imai K (2014). Mediation: R package for causal mediation analysis. J Stat Softw.

[17] Ministry of Health (2019). [Statements to the draft bill of the Measles Protection Act] Stellungnahmen zum Referentenentwurf Masernschutzgesetz.

[18] Ministry of Health (2015). [National Action Plan 2015–2020 for the elimination of measles and rubella in Germany] Nationaler Aktionsplan 2015–2020 zur Elimination der Masern und Röteln in Deutschland.

[19] Robert Koch Institute. [Vaccination coverage at school entry in Germany 2017] Impfquoten bei der Schuleingangsuntersuchung in Deutschland 2017. Epidemiol Bull. 2019;(18):147–53.

[20] Poethko-Muller C, Schmitz R (2013). Vaccination coverage in German adults: results of the German health interview and examination survey for adults (DEGS1). Bundesgesundheitsbl Gesundheitsforsch Gesundheitsschutz.

[21] afp/hil. [Lauterbach wants a new debate on measles vaccine mandate] Lauterbach will Impfpflicht für Masern neu debattieren. Deutsches Ärzteblatt 2019. https://www.aerzteblatt.de/nachrichten/100521/Lauterbach-will-Impfpflicht-fuer-Masern-neu-debattieren. Accessed 14 Feb 2021.

[22] dpa/afp. [Ministry of Health endorses debates on measles vaccine mandate.] Gesundheitsministerium begrüßt Debatte über Impfpflicht gegen Masern. Deutsches Ärzteblatt 2019. https://www.aerzteblatt.de/nachrichten/101897/Ge%C2%ADsund%C2%ADheits%C2%ADmi%C2%ADnis%C2%ADterium-begruesst-Debatte-ueber-Impfpflicht-gegen-Masern Accessed 14 Feb 2021.

[23] Christian Democratic Union of Germany. [Party Convention 2015. Resolutions.] 28. Parteitag der CDU Deutschlands 2015. Sonstige Beschlüsse. 2015; https://www.cdu.de/system/tdf/media/dokumente/sonstige-beschluesse.pdf?file=1 Accessed 14 Feb 2021.

[24] German Ethics Council. Vaccination as a Duty? Opinion. 2019; https://www.ethikrat.org/en/press-releases/2019/ethics-council-increasing-measles-vaccination-rate-by-a-package-of-measures-rather-than-by-mandatory-vaccination/?cookieLevel=accept-all&cHash=5c87d77c3ad2efcd916bb8b9b6a2e751. Accessed 14 Feb 2021.

[25] Omer SB, Betsch C, Leask J (2019). Mandate vaccination with care. Nature..

[26] Betsch C, Böhm R (2015). Detrimental effects of introducing partial compulsory vaccination: experimental evidence. Eur J Pub Health.

[27] Sprengholz P, Betsch C (2020). Herd immunity communication counters detrimental effects of selective vaccination mandates: experimental evidence. EClinicalMedicine..

[28] Schröder-Bäck P, Brand H, Escamilla I, Davies JK, Hall C, Hickey K, Jelastopulu E, Mechtler R, Volf J (2009). Ethical evaluation of compulsory measles immunisation as a benchmark for good health management in the European Union. Cent Eur J Public Health.

[29] Mathieu P, Gautier A, Raude J, Goronflot T, Launay T, Debin M (2019). Population perception of mandatory childhood vaccination programme before its implementation, France, 2017. Eurosurveillance..

[30] Gualano MR, Bert F, Voglino G, Buttinelli E, D'Errico MM, De Waure C (2018). Attitudes towards compulsory vaccination in Italy: results from the NAVIDAD multicentre study. Vaccine..

[31] Böhm R, Betsch C, Korn L (2016). Selfish-rational non-vaccination: Experimental evidence from an interactive vaccination game. J Econ Behav Organ.

[32] Seale H, Leask J, Raina MacIntyre C (2009). Do they accept compulsory vaccination?: awareness, attitudes and behaviour of hospital health care workers following a new vaccination directive. Vaccine..

[33] Association of Statutory Health Insurance Physicians (2019). Statistical information from the Federal Registry of Physicians.

[34] Neufeind J, Betsch C, Habersaat KB, Eckardt M, Schmid P, Wichmann O (2020). Barriers and drivers to adult vaccination among family physicians – insights for tailoring the immunization program in Germany. Vaccine..

[35] Meier NW, Böhm R, Korn L, Betsch C (2020). Individual preferences for voluntary vs. mandatory vaccination policies: an experimental analysis. Eur J Pub Health.

[36] Sprengholz P, Felgendreff L, Böhm R, Betsch C (2021). Vaccination Policy Reactance: Predictors, Consequences, and Countermeasures.

[37] Gualano MR, Olivero E, Voglino G, Corezzi M, Rossello P, Vicentini C, Bert F, Siliquini R (2019). Knowledge, attitudes and beliefs towards compulsory vaccination: a systematic review. Hum Vaccines Immunother.

[38] Gagneur A (2020). Motivational interviewing: a powerful tool to address vaccine hesitancy. Can Commun Dis Rep.

[39] Attwell K, Ward JK, Tomkinson S. Manufacturing consent for vaccine mandates: a comparative case study of communication campaigns in France and Australia. Front Commun. 2021;6(20):598602.

[40] Lewandowsky S, Cook J, Ecker UKH, Albarracín D, Amazeen MA, Kendeou P (2020). The debunking handbook 2020.

