# Supplementary material for: Determinants of physician attitudes towards the new selective measles vaccine mandate in Germany
Source: BMC Public Health. 2021 Mar 22;21:566. doi: 10.1186/s12889-021-10563-9 (PMC7986548; doi:10.1186/s12889-021-10563-9)
Supplement: Supplementary file 1 — Additional file 1: Supplementary Table 1. Title: Mediation analyses: Effect of occupational group (X) on attitude towards mandates (Y) via expected consequences (M). Supplementary Table 2. Title: Sociodemographic characteristics of the study population and of physicians in Germany [file 12889_2021_10563_MOESM1_ESM.pdf]

Determinants of physician attitudes towards the new selective measles vaccine mandate in Germany

Julia Neufeind[1,2], Cornelia Betsch[3,4], Vera Zylka-Menhorn[5], Ole Wichmann[1]

[1]Immunization Unit, Robert Koch-Institute, Berlin, Germany

[2]Charité University Medicine Berlin, Berlin, Germany

[3]Center for Empirical Research in Economics and Behavioral Sciences (CEREB), University of Erfurt, Erfurt, Germany

[4]Media and Communication Science, University of Erfurt, Erfurt, Germany

[5]Deutsches Ärzteblatt, Cologne, Germany

Supplementary Table 1

Mediation analyses: Effect of occupational group (X) on attitude towards mandates (Y) via expected consequences (M).

	Mediator variable model (outcome: expected consequences)			
Predictor	*B*	SE	95% CI	*p*
Constant	2.30	0.33	(1.66-2.95)	
Occupational group	0.50	0.07	(0.39-0.61)	<.001
	Dependent variable model (outcome: attitude towards mandates)			
	Model summary: $R^2 = 0.29$			
Predictor	*B*	SE	95% CI	*p*
Constant	1.10	0.30	(0.52-1.69)	
Occupational group	0.07	0.05	(-0.03-0.17)	.19
expected consequences	0.25	0.02	(0.21-0.29)	<.001
	Indirect effect of X on Y via expected consequences			
Mediator	*B*		95% Quasi-Bayesian CI	*p*
expected consequences	0.13		(0.09-016)	<.001

n = 1,974

Occupational groups: 0 = pediatrician, 1 = other physicians. attitude towards mandates: mean score consisting of four items (Cronbach's alpha = .88) expressing negative attitude (score = 1) to positive attitude (score = 5). Expected consequence of the mandate: item 'I expect more children to be vaccinated on time' (1=disagree; 5=strongly agree). 95% CI: 95% confidence interval with lower and upper border. β coefficients are adjusted for sociodemographic characteristics, communication self-efficacy, patient clientele and 5C psychological determinants.

Supplementary Table 2

Sociodemographic characteristics of the study population and of physicians in Germany[a]

Variable	Level	Pediatrician		GP		Gynecologist		Internist	
		Study population	in Germany	Study population	in Germany	Study population	in Germany	Study population	in Germany
n		376	7,832	1,178	34,554	259	12,611	416	28,732
Gender: n (%)	Male	173 (48.2)	3,493 (44.6)	512 (46.0)	17,908 (51.8)	63 (25.6)	3,992 (31.7)	221 (55.1)	18,403 (64.1)
	Female	186 (51.8)	4,339 (55.4)	601 (54.0)	16,646 (48.2)	183 (74.4)	8,619 (68.3)	180 (44.9)	10,329 (35.9)
Region: n (%)	Western	283 (80.2)	6,193 (79.1)	895 (81.4)	27,837 (80.6)	184 (75.7)	10,090 (80.0)	321 (80.2)	22,576 (78.6)
	Eastern	70 (19.8)	1,639 (20.9)	205 (18.6)	6,717 (19.4)	59 (24.3)	2,521 (20.0)	79 (19.8)	6,156 (21.4)

[a] Statistical Information from the Federal Registry of Physicians, 2019
